# Determining Model for Maximum Blood Request(MSBOS) for Surgery: An Elective Surgery in Imam Ali Hospital, Zahedan, Iran

**Published:** 2019-04-01

**Authors:** Seyed Mehdi Hashemi, Seyed Hossein Soleimanzadeh Mousavi, Zeinab Tavakolikia

**Affiliations:** 1 Department of Internal Medicine, School of Medicine, Imam Ali Hospital Complex, Zahedan University of Medical Sciences, Zahedan, Iran; 2 Deprtment of Pediatric, School of Medicine, Imam Ali Hospital Complex, Zahedan University of Medical Sciences, Zahedan, Iran; 3 General Practitioner, Zahedan University of Medical Sciences, Zahedan, Iran

**Keywords:** MSBOS, Blood transfusion, Blood transfusion pattern

## Abstract

**Background:** Nowadays, excessive blood intake is one of the most common problems in educational hospitals, causing issues such as the lack of proper distribution of blood products among centres, increases in costs and blood bank workloads. So, programs such as a Maximum Surgical Blood Ordering Schedule (MSBOS) were introduced to design a blood ordering schedule, which is a guide to normal transfusion needs for common surgical procedures.

**Materials and Methods:** This study was a descriptive cross-sectional study. The sampling method was designed and distributed among all sectors of the hospital. Each sector according to the demand for blood and cross-matched transfused units entered the rate of wasted and unused blood bags on the related forms. This study was performed on 1568 patients, of whom 562 (35/84%) were given blood transfusions.

**Results:** The aim of this study was to determine the pattern for the maximum surgical blood order schedule (MSBOS) for elective surgical procedures/in elective surgery cases in Imam Ali Hospital, Zahedan. This study was performed on 1568 patients, of whom 562 (35/84%) were given blood transfusions. The mean C/T ratio was 1.61 ± 0.99, the mean TI was 0.61 ± 0.38, and the mean T index was 36.4 ± 30.16%.

**Conclusion: **In general, only 55% of the blood units were used. Hernia surgery, thyroidectomy, and patients with renal problems had the greatest number of wasted units. Therefore, according to the results, indications of blood donation should be made correctly by health care personnel in all patients requiring a blood transfusion, and if there is an increased number of indications, packed cells are requested.

## Introduction

 Nowadays, excessive blood intake is one of the most common problems in educational hospitals, causing issues such as poor distribution of blood products among centres, increases in costs and blood bank workloads^1^^,^^2^. This high demand is due to fears of inadequate access to adequate blood supply during surgery and or due to the lack of a specific pattern for requesting blood that ultimately worsens the overall condition and causes a delay in all surgical procedures^3^. 

Various studies have shown that non-observance of the principles of blood demand for surgery leads to false deficiencies, increased age of blood stored in the blood bank, reduced quality and imposes heavy laboratory costs, and increases waste due to the expiration of the date of consumption. The side effects of unnecessary blood transfusions and its products are likely to cause contamination in the blood recipient^4^. Most studies in the 70s and 80s have shown the excessive demand for blood and blood products and their lack of use in many surgical procedures^5^^-^^7^. So, Maximum Surgical Blood Ordering Schedule (MSBOS) for surgery was designed based on the reports from each hospital about the amount of blood used during surgical operations of various types, providing a special guide for each centre to list the number of units of blood routinely requested. Based on the pattern, the request for an adaptation test will be limited to a number of surgical procedures, which have a high risk of blood transfusion^4^^,^^8^. The request for blood in these surgeries, based on the consumption pattern of each surgeon, set at the level, indicating the actual consumptions in each surgery. So, it leads to a reduction in the number of compatibility tests, intact blood products in most cases, and the effective use of the blood inventory ^11^^-^^13^. Some studies conducted in recent years have reduced the number of cross-match cases. Half of the cases were identified before MSBOS, and considerable saving was made in costs and resources.^1^^, ^^2^^,^^8^^,^^14^

**Figure:**
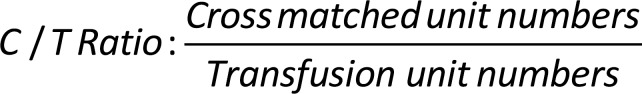


In the year 1975, Henry and Burol proposed C/T Ratio (Crossmatch/Transfusion) and TI (Transfusion Index) to examine how demand and blood consumption were in the hospitals. The C/T index is the ratio of blood cross-match blood units to the number of injected blood units.

**Figure:**
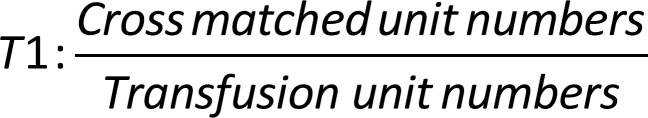


Researchers rated 2.1 to 3.1 as the result of the optimal use of blood. In other words, in the management of blood transfusions, the C/T ratio, if greater than 2.5, indicates that less than 40% of the cross-match units are consumed in blood transfusions.

The TI index is referred to the blood transfusion index and represents the number of units injected per number of cross-match units.^9^^,^^10^

**Figure:**
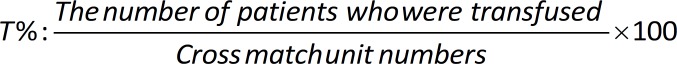


TI of more than 0.5 indicates a significant need for blood during surgery.

The T-index of the number of patients undergoing blood transfusion is expressed as the percentage of patients who are cross-matched^9^^,^^10^. 

**T**% of more than 30 indicates a significant need for blood during surgery. The aim of this study was to determine the MSBOS pattern for all surgical procedures in Imam Ali Hospital, Zahedan. It is the first study of its kind in this centre which was designed to reduce the waste of blood bags and save our country's monetary resources.

## MATERIALS AND METHODS

 This is a descriptive cross-sectional study in which all patients referred to Imam Ali Hospital for elective surgery during from September 2016 to September 2017. The sampling method was designed and distributed among all sections of the hospital. Each section, according to the blood demand and cross-matched and blood transfusion rate statistics, determined the wasted and unused blood bag and register it in the appropriate forms. Finally, these forms were collected and after the information was recorded in the unit form, we extracted and used all the information.

Descriptive statistics Tables, percentages, Tables and charts were used for data analysis. At the end of the study, the data were entered into SPSS software and analyzed. To compare the variables in both groups, t-test, chi-square and Fisher’s exact test or U-Mann Whitney test were used.

This is a descriptive cross-sectional study in which all patients referred to Imam Ali Hospital for elective surgery between September 2016 and September 2017. The sampling method was designed and distributed among all hospital sectors. Each sector according to the demand for blood and cross-matched transfused units entered the rate of wasted and unused blood bags on the related forms. Finally, these forms were collected, and all data were recorded on the unit form to extract meaningful information. Descriptive statistics Tables, percentages, Tables and charts were used for data analysis. At the end of the study, the data were entered into SPSS software and analyzed. To compare the variables in both groups, t-test, chi- square and Fisher’s exact test or U-Mann Whitney test were used.

## Results

 The aim of this study was to determine the pattern for maximal request for surgical surgeries (MSBOS) in elective surgery (elective) in Imam Ali Hospital, Zahedan. This study was performed on 1568 patients, of whom 562 (35/84%) were given blood transfusion. The findings of this study are presented in the following Tables (Tables 1 & 2).

**Table 1:** Mean and standard deviation of blood transfusion indices in the population under study

Group	Minimum	Maximum	Average	Standard deviation
Requested Blood units	1	760	33.96	91.77
Cross-match units	0	620	28.98	75.32
Blood injected Unit	0	489	18.98	57.28
C / T Ratio	1	6	1.61	0.99
TI Index	0	1	0.61	0.38
T Indicator	0	100	36.04	30.16

As shown in the Table 1, the average C / T ratio was 1.61 ± 0.99, the mean TI was 0.61 ± 0.38 and the mean T index was 36.4 ± 30.16%. 

As shown in the Table 2, the highest number of blood units were requested for patients under CABG (760 units), caesarean section (243 units) and hysterectomy (150 units). The lowest number of blood units were requested for patients with other complaints (4.35 units), perineal rupture (12 units) and nephrectomy (14 units). The highest number of crossed units were requested for patients under CABG (620 units), caesarean section (202 units) and hysterectomy (132 units). The lowest number of crossed units were requested for patients with other complaints (4.07 units), perineal rupture (12 units) and nephrectomy (8 units). The highest number of injected units was seen in patients under CABG (489 units), caesarean section (87 units) and hysterectomy (86 units). The lowest number of injected units were seen in patients with ventricular fibrillation (0 units), neck mass (0 units) and prostate hypertrophy (0 units). The highest C / T ratio was seen in patients with Hernia (6), thyroidectomy (4.5), and renal problems (3.4%). The lowest C / T ratio was observed in patients with gastric cavity (1), tamponade (1), and vaginal rupture (1). The highest TI was seen in patients with gastric carcinoma (1), tamponade (1) and vaginal rupture (1). The lowest TI was found in patients with interstitial ventricular defects (0), neck mass (0), and prostate hypertrophy (0). The highest T index was in patients with splenectomy (66.66), fistula (63.15) and femoral fracture (58.33). The lowest T index was in patients with ventricular fibrillation (0), neck mass (0) and prostate hypertrophy (0).

**Table 2:** The mean of the variables studied based on patients' problems

Complaints patients need injection	Required blood unit	Cross-Matched blood unit	Blood unit injected	C / T Ratio	TI indicator	T index
CABG	760	620	489	1.26	0.78	13.38
Renal problems	17	17	5	3.4	0.29	17.64
Thyroidectomy	20	18	4	4.5	0.22	11.11
Bed Sore	52	49	42	1.16	0.85	51.02
Splenectomy	31	27	24	1.12	0.88	66.66
Esophageal Cancer	36	35	23	1.52	0.65	34.28
Rupture of the vagina	21	19	19	1	1	21.05
Caesarean section	243	202	87	2.32	0.43	13.86
Curettage	83	75	43	1.74	0.57	24
Bladder tumor	38	36	25	1.44	0.69	44.44
Femoral fracture	28	24	23	1.04	0.95	58.33
Laparotomy	27	18	17	1.05	0.94	55.55
Hysterectomy	150	132	86	1.53	0.65	18.18
Colon Cancer	43	40	23	1.73	0.57	30
Nephrectomy	14	8	3	2.66	0.37	12.5
Prostate hypertrophy	24	21	0	-	0	0
Abdominal Pelvic Resection	24	22	9	2.44	0.4	22.72
Natural delivery	31	26	23	1.13	0.88	30.76
Restoration	46	41	23	1.78	0.56	34.14
Myomectomy	30	30	15	2	0.5	20
Amputation	61	53	29	1.82	0.54	26.41
Trauma	88	85	63	1.34	0.74	44.7
Biliary Surgery	114	109	41	2.65	0.37	22.01
Fistula	19	19	15	1.26	0.78	63.15
Haematuria	21	21	17	1.23	0.8	57.14
Perineal tear	12	12	10	1.2	0.83	25
Herniation	18	18	3	6	0.16	11.11
Neck mass	16	12	0	-	0	0
Mitral Valve Problems	58	43	34	1.26	0.79	48.83
TOF	25	17	16	1.06	0.94	47.05
Aortic aneurysm	83	30	29	1.03	0.96	46.66
Aortic valve replacement	21	19	15	1.26	0.78	42.10
Tamponade	26	25	25	1	1	44
Stomach Cancers	26	25	25	1	1	48
Flap	74	63	54	1.16	0.85	38.09
Ectopic pregnancy	27	27	21	1.28	0.77	40.74
Mole	20	20	7	2.85	0.35	15
Ventricular wall defect	18	15	0	-	0	0
Other	4.35	4.07	1.9231	1.37	0.58	40.52
Total	33.96	28.98	18.9870	1.61	0.61	36.04

**Table 3:** Mean and standard deviation of blood transfusion indices in the population under study

Variable	Section	Average	Standard deviation	P-value
Required blood unit	Surgery	32.16	97.77	0.302
	Obstetrics	40.81	66.12	
Cross-matched unit	Surgery	27.13	79.97	0.26
	Obstetrics	36.06	55.66	
Blood unit injected	Surgery	18.6	62.9	0.165
	Obstetrics	20.43	28.15	
C / T Ratio	Surgery	1.56	1	0.288
	Obstetrics	1.75	0.97	
TI indicator	Surgery	0.6	0.39	0.853
	Obstetrics	0.64	0.31	
T index	Surgery	39.26	32.38	0.076
	Obstetrics	22.98	12.28	

Mann-whitney u test

As shown in the Table above, the mean and standard deviation of blood transfusion indices in the studied population were not statistically significant difference (P> 0.05).

## Discussion

 The aim of this study was to determine the pattern for maximal request for surgical surgeries (MSBOS) in elective surgery (elective) in Imam Ali Hospital, Zahedan. This study was performed on 1568 patients, of whom 562 (35/84%) were given blood transfusion.

In a study conducted by Soleimanha et al. in 2015 at Pursina Hospital, Rasht, among 872 orthopaedic patients selected for surgery, 318 of them were cross-matched and only 114 of them were given blood transfusion. In this study, C / T Ratio was 6.4, the probability of transfusion (T %) was 36.47%, and the transfusion index (TI) was 0.9, indicating that blood transfusion requirements were somewhat higher than standard^15^. In our study, we found that the mean C / T ratio was 1.61 ± 0.99, the mean TI Was equal to 0.61 ± 0.38 and the mean T index was 36.40 ± 30.16. Compared to Soleimanha et al.’ s study, only T-index was similar to their results, and the C / T ratio and the transfusion index were significantly better in our study. This difference is due to the type of collision in the consumption of blood and demand for blood in our study compared to Pursina Hospital in Rasht. Meanwhile, other causes of the difference may be due to the difference in the sample size, inclusion and exclusion criteria and sampling method.

Another study was conducted by Khalili Aalam et al. in Firoozgar Hospital in Tehran. This retrospective study consisted of 364 patients selected for 6 elective surgeries (cesarean section, thyroidectomy, total hysterectomy, laminectomy, cholecystectomy and mastectomy) in 2002. Necessary information was obtained, and with regard to C / T ratios, T% and TI, a program was proposed for these surgeries. Due to the high ratio of C / T (21.5) and low percent of cross-linked blood (4.7%), it was necessary to change the pattern of blood demand in this centre, so, the Type and Screen (T&S) method was suggested to use before these procedures^16^. However, in our study, the mean C / T ratio was 1.61, the mean TI was 61.0 and the mean T-value was 36.04%. The C / T ratio and transfusion index were significantly better in our study.

In another study by Hasan Rafiei in Besat Hospital in Hamadan (2009), among 926 blood samples obtained from males (63%) and females (37%) with a mean age of 28 years, the overall C / T and TI index were 2.01 and 0.86, respectively. Compared to the standard number (C / T <2.5 and TI> 0.5), the target outcome was is in desirable condition^17^. Compared to our study, it was found that in our study the indicator C / T is more favourable, but the TI index is significantly lower than that of the study, which indicates that our condition is worse. Therefore, although the findings of our study indicate a favourable situation (C / T <2.5 and TI> 0.5). 

In a descriptive study by Hayedeh Ala'a al-Dawlah et al. at Yahyanejad Hospital in Babylon, the consumption of blood was assessed among 1043 patients during three months. The results of the study showed the highest C / T ratio in the elective section (14.7) with TI = 0.16, indicating that the required blood count was about 15 times more than the blood transfusion^9^. Meanwhile, in our study, the requested blood was about 1.6 times more than the blood injected, which is much more favourable. This difference is due to the type of collision in the blood consumption and blood demand in our study hospital and Pursina Hospital in Rasht. Meanwhile, other causes of the difference may be due to the difference in the sample size, inclusion and exclusion criteria and sampling method.

Gharehbaghan et al. conducted a cross-sectional descriptive study on the blood request forms of patients (n=491) in five hospitals in Rasht (2007). As results shows, the highest and lowest blood request were received from Departments of Surgery and Internal Medicine, respectively and the C / T index was 1.9^10^. The results of this study were in line with the findings of our study.

In another descriptive study carried out by Dr. Behrooz Zaman et al. in Rasoul-e-Akram Hospital in Tehran, only 1460 blood bags (17.4%) from the 8385 total blood bags reserved for elective surgery were injected. The blood cross-threaded blood injection rate was 3.12. The main cause of wasting blood was the error made by surgical groups (44.6%) ^18^. However, in our study, the blood cross-matched to transfusion ratio was calculated as 1.61 (half of the study results) and it turned out that the estimated mistake in obstetrics surgery was more than other surgical procedures (1.75 versus 1.56), but it was not statistically significant

Different studies have also been conducted in other countries. In England, in May 1996, the C / T ratio was 2.2 in general surgery and 2.3 in orthopaedic^19^^, ^^20^, while it was 1.61 in our study, which is far better than the above-mentioned study. Moreover, the highest C / T ratio was found in patients with hernia (6), thyroidectomy (4.5), renal problems (3.4) and orthopaedic surgeries, including femoral fractures (1.04). Therefore, the C / T ratio in general surgery is much higher than orthopaedic surgeries, which is different from the outcome of the study.

Between 1999 and 2001, London University Hospitals implemented the MSBOS program in the surgical ward and the C / T ratio decreased from 2.25 to 1.71, resulting in saving 102 blood units 15 months.^21^ In our study, the C / T ratio was 61/1, but careful consideration would need to be given in requesting and setting up a flowchart to carefully examine the indication of blood reserve for a hospital in various surgeries to reduce the loss of blood.

In the Shah Fahad University Hospital in Saudi Arabia, during the 4-year program (a type and screen program), the C/T ratio decreased from 4.8 to 2.1^22^. Therefore, it is possible to carefully answer the request and make a flowchart for accurate selection and indication before blood is requested. 

In Sistan and Baluchistan, Zahedan is a specialist medical centre. Therefore, demand for blood, the existence of a program to supply blood and reduction of its costs are among the most notable cases in the hospitals of this city. According to the data extracted from the Transfusion Centre in Zahedan (2016), the total number of blood bags not used in all Zahedan hospitals was 882 bags and about USD 120 million (considering the cost per blood bag is about 134000 USD) was wasted. According to the statistics from the organization, the most blood bags were wasted in Imam Ali Hospital (283 bags of blood) and the least blood bags were wasted in Booali Hospital (4 bags of blood). The most wasted blood was released in the September and the lowest in the month of July.

In a retrospective study conducted by Dr. Karami et al. in the Blood Transfusion Organization in Zahedan, during a 3-month period from August 2006 to October 2007, 2094 blood transfusions ordered by the blood bank of the Zahedan hospitals were reviewed; of which a total of 1536 PC units (44%) were not consumed (677 units)^ 23^. In our study, only 18.98 units of 33.96 units requested were injected (55.88%), indicating that nearly 35% of the requested blood was wasted. Although it is a much more favourable result, careful examination is essential to preserve blood supplies from being wasted in various surgeries and conditions.

## CONCLUSION

 The results of our study showed that the mean C / T ratio was 1.61 ± 0.99, the mean TI was 0.61 + 0.38 and the mean T index was 36.4 ± 30.16%. In general, only 55% of the blood was used. Hernia surgery, thyroidectomy, and patients with renal problems had the most non-used blood bags. Therefore, according to the results, it is suggested that health care personnel make indications appropriately for all patients requiring blood transfusion, and if there is increased number of indications, blood is requested. Meanwhile, setting up flowcharts and making careful query to accurately check the indication of the blood reserve in various surgeries may be helpful in reducing blood loss.

## SUGGESTIONS

According to the results of this study and other studies that showed high demand for blood and the lack of use of 35% of the requested blood (although our study was in desirable areas), the following are suggested:

1. Quick and timely identification of patients with a clear indication of blood transfusion.

2. Holding meetings and presenting information to doctors to introduce strict indications of blood donation in patients with trauma and patients requiring surgery (especially elective surgery such as hernia, thyroidectomy, etc.).

3. Given a small number of studies, studies similar to those of a larger sample are suggested.

4. To conduct a systematic study in line with the objectives of the present study.

5. Also, the results of this study will introduce more comprehensive research based on the wider sample with the above mentioned issues. If the results of this research are confirmed by larger studies, they will be compared to the results of the other community of physicians using appropriate screening methods in patients in need of blood transfusions. Considering the sensitivity of the treatment of disease and limitations of this study such as the lack of a larger sample size, we suggest that a more prospective study be designed based on available information and using the most efficient statistical methods to provide more comprehensive information. 
